# Synthesis of Resorcinol-Based Phosphazene-Containing Epoxy Oligomers

**DOI:** 10.3390/polym11040614

**Published:** 2019-04-03

**Authors:** Igor A. Sarychev, Igor S. Sirotin, Roman S. Borisov, Jianxin Mu, Irina B. Sokolskaya, Julya V. Bilichenko, Sergey N. Filatov, Vyacheslav V. Kireev

**Affiliations:** 1 Mendeleev University of Chemical Technology of Russia, Miusskaya sq. 9, 125047 Moscow, Russia; yahoo123-92@mail.ru (I.A.S.); julyab2@gmail.com (J.V.B.); filatovsn@muctr.ru (S.N.F.); kireev@muctr.ru (V.V.K.); 2 Topchiev Institute of Petrochemical Synthesis, Russian Academy of Sciences, Leninskii pr. 29, 119991 Moscow, Russia; borisov@ips.ac.ru; 3 Peoples’ Friendship University of Russia, Miklukho-Maklaya str.6, 117198 Moscow, Russia; 4 College of Chemistry, Jilin University, 2699 Qianjin Street, Changchun 130012, China; emujianxin@163.com; 5 State Research Institute of Chemistry and Technology of Organoelement Compounds, sh. Entuziastov 38, 111123 Moscow, Russia; irina-sokol@list.ru

**Keywords:** phosphazene, cyclophosphazene, epoxy resin, epoxy oligomers, resorcinol

## Abstract

Phosphazene-containing epoxy-resorcinol oligomers (PERO) are synthesized in one stage with the direct interaction of hexachlorocyclotriphosphazene (HCP), resorcinol, and epichlorohydrin in the presence of solid NaOH. Depending on the initial ratio of HCP:resorcinol, PERO contains from 20 to 50 wt.% phosphazene component (2.0–4.8% of phosphorus) and have an epoxy group content up to 30 %. Products are characterized using ^1^H and ^31^P NMR spectroscopy, MALDI-TOF mass spectrometry, and elemental analysis. According to mass spectrometry, the phosphazene fractions of PERO include up to 30 individual compounds with a predominance of cyclotriphosphazenes with one unsubstituted chlorine atom and four or five glycidyl groups. PERO has a lower viscosity in comparison with similar resins based on bisphenol A, which can simplify their use as a binder for polymer composites, adhesives, and paints.

## 1. Introduction

Epoxy resins have won a special place in industry and everyday life—though not so much in terms of production, but in their specific role [1]. Classic bisphenol A based epoxy resins have many advantages, including good mechanical and adhesive properties [1,2], which have led to their indispensability as the basis of adhesives, coatings and binding polymer composites [1,3]. However, polymers based on neat epoxy oligomers often have detracting characteristics, in particular, flammability and relatively low heat resistance [3].

A rather effective way to increase the heat resistance of epoxy matrices is their structural modification, in particular, with compatible oligomers with higher functionality, which can be incorporated into the three-dimensional network structure formed during curing [3,4]. Reducing the flammability of epoxy polymers is a more difficult challenge. Regarding materials based on brominated epoxy resins, as well as other halogen-containing compounds, although they contribute to the reduction of flammability, they can emit toxic gases when in contact with a flame, limiting their use for environmental reasons [5,6]. One of the most promising methods of modifying epoxy polymers in order to impart heat and fire resistance is the introduction of phosphorus compounds, known as universal flame retardants for a large number of polymeric materials. Phosphorus helps reduce flammability, and when exposed to flame, significantly fewer toxic substances are released. The current global trend is the rejection of halogen-containing fire-resistant materials in favor of safer phosphorus-based [5]. However, the use of phosphorus-containing additive type flame retardants is associated with some difficulties. Thus, phosphoric esters can act as plasticizers, reducing the heat resistance of the material [7]; they are also poorly compatible with epoxy polymers [8,9]. In addition, most industrial phosphorus-containing flame retardants have a relatively low degradation temperature and limited chemical resistance [5,6,10,11].

One of the promising classes of compounds that can be used as modifiers that reduce the flammability of polymers are phosphazenes [6,12,13]. The main chain of organophosphazenes consists of alternating atoms of phosphorus and nitrogen, and at the phosphorus atom, there are organic radicals introduced by the substitution of halogen in halogenphosphazenes [14]. The nature of organic substituents can vary widely and defines the properties of the final polymer or oligomer. [14]. Aryloxyphosphazenes, for example, in comparison with other phosphorus-based flame retardants have, as a rule, higher thermal stability and chemical resistance [6,11,13,15]. Organophosphazenes are effective flame retardants. The introduction of commercially available phosphazene-based flame retardant hexaphenoxycyclotriphosphazene [13,16] in polyamide-6 also contributes to an increase in its mechanical characteristics [17]. Propyl ester phosphazene significantly increases the temperature of the onset of decomposition and the char yield of polymethyl methacrylate [18] and polystyrene [19].

In order to avoid possible negative effects arising from the use of additive type flame retardants, it is preferable to ensure the formation of covalent bonds of the flame-retardant molecules with the matrix of the modified polymer. Therefore, halogenphosphazenes, of course, are of interest as a universal, flexible building block, which makes it possible to easily obtain functional organophosphazenes as well.

Phosphazenes with reactive functional groups in an organic substituent, when introduced into thermosetting matrices, perform not only the function of a flame retardant, but can also change the structure of the three-dimensional network and positively affect the performance properties of the polymer. Thus, functional phosphazenes increase fire resistance [20], thermal stability, char yield [21], and tensile strength [22] of polyurethanes, as well as hardness and hydrophobicity of coatings based on them [23]. Self-crosslinking acrylic latex, which was obtained via an emulsion polymerization of 2,2,2-trifluoroethyl methacrylate, methyl methacrylate, butyl acrylate, methacrylic acid, and hexakisallylaminocyclotriphosphazene, exhibited improved stability, physico-chemical, electrochemical and properties [24].

Epoxy resins modified with epoxyphosphazene are characterized by low flammability, increased heat resistance and char yield, hydrophobicity, and non-toxicity of combustion products [25,26,27,28,29,30,31], enhanced mechanical characteristics and dielectric properties at the level of basic epoxy resins [32].

Currently, there are 2 main synthetic approaches that allow obtaining functional phosphazenes capable of forming covalent bonds with epoxy matrices: synthesis of organophosphazenes with reactive epoxy groups [25,26,27,33,34,35,36,37,38,39,40,41,42,43,44,45,46,47,48,49,50,51,52,53,54,55] for addition to the epoxy component, and synthesis of organophosphazenes with reactive amine [56,57,58,59,60,61,62,63,64] groups for use as a hardener. However, at present, most of the processes of synthesis of functional epoxyphosphazenes described in the literature are primarily of scientific interest because of the complexity of their scaling and the large number of intermediate stages [25,33,34,35,36,37,41,42,43,45,46,47,48,50,51,52,53,54,55].

In Kireev et al.’s study [65], several methods for the synthesis of phosphazene-containing epoxy oligomers (PEO) by oxidation of eugenol derivatives of cyclophosphazenes and epoxidation of hydroxyaryloxycyclophosphazene (GARP) with epichlorohydrin were compared. Synthesis of the original GARP by the interaction of chlorocyclophosphazenes with diphenols is complicated by the high functionality of the system and the need to use a significant excess of diphenol [66,67], the subsequent separation of which is a difficult, time-consuming task. Therefore, a method for epoxidizing an unseparated mixture of GARP and excess of diphenol was developed [68].

The simplest, most convenient and promising method for obtaining PEO—based on hexachlorocyclotriphosphazene (HCP) and bisphenol A—is a one-step one-pot process of the interaction of these monomers in the presence of an alkaline agent in an environment of excess epichlorohydrin as a reagent and solvent [69,70].

Most of the above-described phosphazene epoxides are solids or highly viscous liquids that can reduce the processing properties of modified epoxy resins and make it impossible to process them at room temperature.

Thus, the development of new simple methods for obtaining of aryloxyphosphazene-containing epoxy resins—based on readily available, inexpensive starting reagents with reduced viscosity and improved processed properties—is of great scientific and practical interest and may contribute to accelerating the widespread use of phosphazene-containing resins as components of high-tech polymer composite materials.

**Figure 1:**
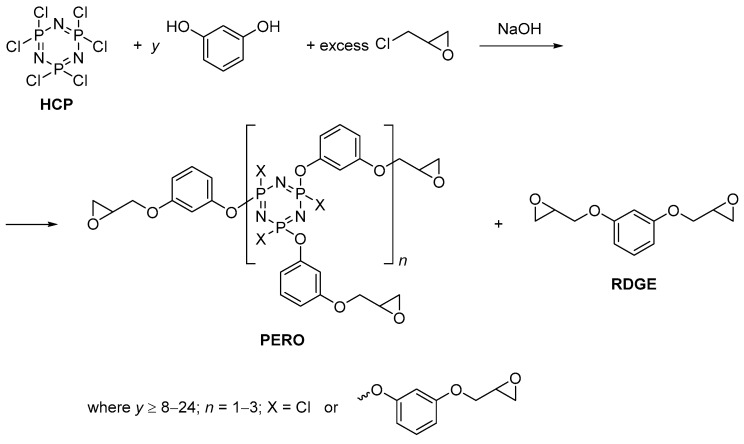
The synthesis of phosphazene-containing epoxy-resorcinol oligomers (PERO).

In order to obtain a low viscosity PEO with greater phosphorus content in the present work, instead of bisphenol A, resorcinol was used, the interaction of which with HCP and epichlorohydrin was performed according to the scheme in Figure 1.

## 2. Materials and Methods

### 2.1. Starting Materials

Hexachlorocyclophosphazene—a white crystalline substance with m.p. of 113 °C; NMR ^31^P-singlet spectrum with δ_P_ = 19.9 ppm, was obtained by the method [71].

99.5% pure (according to the quality declaration) sodium hydroxide (NaOH) from JSC KAUSTIK (Volgograd, Russia), in white granules with a diameter of 1–2 mm was used without purification. The content of crystallization water determined by acid-base titration was about 5%.

Epichlorohydrin (Solvay) with the content of the main substance of 99.8% was distilled before use, b.p. 118 °C.

Solvents were purified according to known methods, their physical characteristics corresponded to the literature data [72].

The viscosity of the PERO obtained was compared with D.E.R. 332 epoxy resin (Dow Chemical, Midland, TX, USA) with the epoxy group content of 24.6–25.1% and epoxy equivalent of 171–175.

### 2.2. Synthesis of Epoxyphosphazenes

In order to adjust the content of the phosphazene component in the product, a series of experiments was carried out according to the methods below, changing the amounts of the initial reagents in accordance with the Table 1. The alkali was taken in a 5% molar excess relative to the number of phenolic groups of resorcinol.

#### 2.2.1. Synthesis of Phosphazene-Containing Resorcin-Based Epoxy Resin with the Introduction of the Entire Amount of Alkali at the Beginning of the Reaction

In a four-necked flask with a volume of 250 mL, equipped with an overhead bladed stirrer, distilling trap, and thermometer, 1.00 g (2.87 mmol) HCP, 5.06 g resorcinol (46.0 mmol) and 100 mL epichlorohydrin were charged. The mixture was heated in an oil bath until the reagents were completely dissolved and the epichlorohydrin boiling point was reached. Then, with vigorous stirring, 3.86 g (96.6 mmol) of granulated sodium hydroxide was immediately charged. The reaction was conducted for 30 min, maintaining the boiling of the reaction mixture and distilling off the azeotropic mixture of epichlorohydrin with evolving water. Then the flask was cooled to room temperature, and the epichlorohydrin was distilled off in vacuum at a temperature not higher than 90 °C. The contents of the flask were extracted with 200 mL of acetone, and the formed sodium chloride and possible excess alkali were filtered off. The precipitate was extracted with another 200 mL of acetone, the solvent was distilled off from the combined solutions, and the residue was dried in vacuum at 90 °C until constant weight. 7.04 g (69%) of a viscous transparent light-yellow product with epoxy group content of 22.2% was obtained.

#### 2.2.2. Synthesis of Resorcinol-Based Phosphazene-Containing Epoxy Resin with the Gradual Loading of Alkali

Synthesis of resorcinol-based phosphazene-containing epoxy resin with the gradual loading of alkali was carried out as above and with the indicated amounts of the initial reagents, with the exception that all the calculated amount of alkali was divided into 6–10 equal portions of approximately 0.5 g and added with an interval of 3 min after which the reaction was carried out for 30 min, maintaining the boiling of the reaction mixture and distilling off the azeotropic mixture of epichlorohydrin with evolving water. The product was isolated in the same manner as above.

9.08 g (89%) of a viscous transparent light-yellow product with epoxy group content of 28.6% was obtained.

### 2.3. Methods of Analysis

The ^31^P and ^1^H NMR spectra were measured in chloroform-d solutions with a Bruker AV-400 spectrometer (Bruker Corporation, Bremen, Germany) operating at 162 and 400 MHz, respectively. The signals due to the deuterated solvents were used as internal references. The chemical shifts of the signals were calculated relative to the signals of tetramethylsilane (^1^H) and phosphoric acid (^31^P), which were used as references. The spectra were processed with the help of the MestReNova Lab software package (Version 12.0.3, MESTRELAB RESEARCH, S.L, Santiago de Compostela, Spain).

MALDI-TOF mass spectrometric analysis was carried out on the Bruker Auto Flex II instrument (Bruker Corporation, Bremen, Germany).

Elemental analysis was carried out by spectrophotometry.

In the present paper, epoxy group content (in wt.%) is the weight of oxirane groups (–CHCH_2_O) divided by the total molecular weight of epoxy resin. Thus, the theoretical content of epoxy groups was calculated by the formula:

(1) $$E_{calc}=\frac{43\times n}{M}100\%$$

where 43 and *M* are the molecular weights of the oxirane group and the whole epoxy resin molecule, respectively, and *n* is the number of oxirane groups in the epoxy resin molecule. The experimental epoxy group content was determined by the method of reverse acid-base titration [73], similar to Russian standard GOST 12497-78. In these methods, glycidyl groups are converted to chlorohydrin groups by dissolving the sample of epoxy resin in hydrochloric acid acetone solution followed by titration of the excess of hydrochloric acid with NaOH solution. The experimental epoxy group content is calculated by the formula:

(2) $$E_{exp}=\frac{\left(V-V_{0}\right)\times 43\times N}{g\times 1000}100\%$$

where *V* is the amount of NaOH solution for titration of a sample containing epoxy resin in mL; *V*_0_ is the amount of NaOH solution for titration of blank sample without epoxy resin in mL; 43 is the molecular weight of the oxirane group; *N* is the concentration of NaOH solution, mol∙L^−1^; and *g* is the mass of epoxy resin sample in g.

The hydroxyl group content was determined by the method [74].

The viscosity of epoxy oligomers was evaluated on a Reotest-2 rotational viscometer with a working cone-plane unit.

## 3. Results and Discussion

Previously, using the example of a bisphenol-A based PEO, it was shown that to eliminate gelation in highly functional A_2_ + B_6_ systems (diphenol:HCP), it is necessary to use diphenol in an amount that provides at least a twofold excess of OH-groups relative to P–Cl bonds [69,70].

From Table 2 it follows that at the optimum synthesis temperature (118 °C), duration of 30 min, and single-moment charging of the entire amount of alkali, the yield of PERO is 50–75% and depends little on the HCP:resorcinol ratio, while the epoxy group content increases with the specified ratio due to the increased content of the organic component in PERO (epoxy group content of resorcinol diglycidyl ether is 39%). However, with the gradual addition of alkali (Table 3) for the same ratios of HCP:resorcinol and other conditions being equal, the yield and epoxy group content of PERO formed are much higher (experiments No. 1 and No. 12 or No. 6 and No. 13).

**Figure 2:**
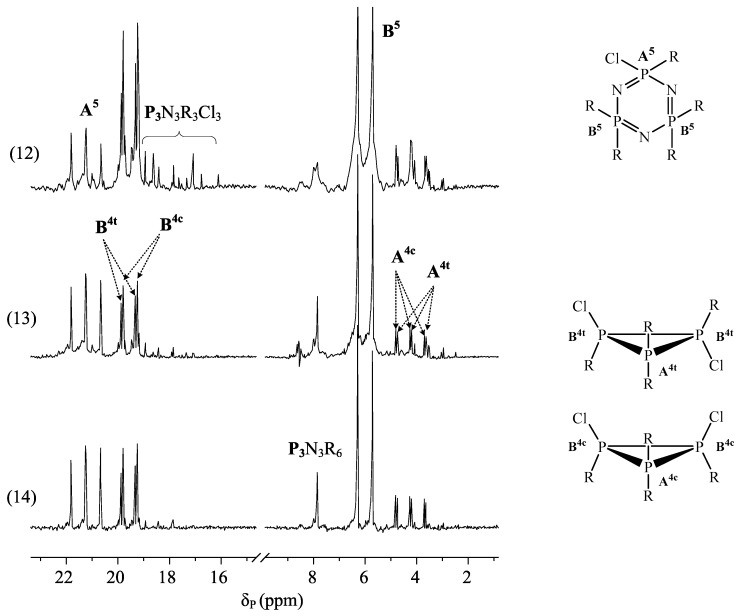
NMR ^31^P spectra of PERO No. 12–14 (Table 3).

As in the case of bisphenol A based epoxyphosphazene oligomers [69,70], the PERO phosphazene fraction mainly contains compounds with 1–2 unsubstituted chlorine atoms in the triphosphazene cycle. This is indicated by the presence in the ^31^P spectra (Figure 2) of signals of phosphorus atoms belonging to tetra- (AB_2_ system with cis-trans isomerism, δ_P_ = 19.4 ppm (dd) and 4.2 ppm (tt)) and penta-substituted (AB_2_ system, δ_P_ = 21.2 ppm (t) and 6.2 (d)) trimeric cycles. The low-intensity singlet signal at 7.9 ppm in the ^31^P NMR spectra indicates an insignificant content of hexa-substituted triphosphazene compounds in PERO.

Determination of the optimal temperature and duration of the process was carried out at the HCP:resorcinol ratio of 1:16, at which the calculated phosphorus content in the product is 3.0% and an acceptable level of fire resistance is reached. Experiment number 2 was carried out in accordance with the terms of the patent [75]: the reaction temperature was 65 °C, time was 90 min, and there was a single-time loading of the alkaline agent. These conditions are optimal if the diphenol is bisphenol A; however, in the case of resorcinol, the reaction mixture is blackened, the yield is low, and the product has a high viscosity. A similar picture is observed at 90 °C (experiment No. 3). Regardless of the HCP:resorcinol molar ratio, the boiling point of epichlorohydrin (118 °C) and a 30-min process duration are the optimal conditions to achieve an acceptable yield of the resulting PEO. After 30 min from the start of the reaction, the nature of the NMR ^31^P and ^1^H spectra of the reaction mixture do not change.

**Figure 3:**
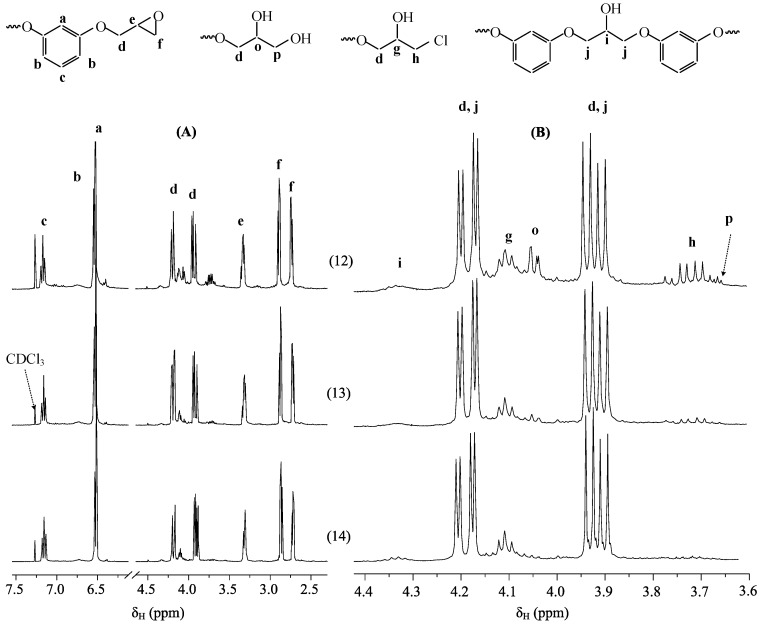
^1^H-NMR spectra PERO No. 12, 13 and 14 (Table 3) with the assignment of signals of protons of different groups (**A**) and an enlarged area with a chemical shift 3.6–4.4 ppm (**B**).

**Figure 4:**
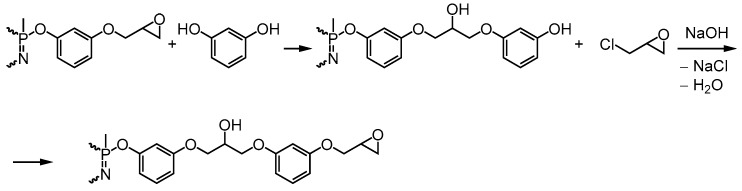
Side chain oligomerization in epoxyphosphazene.

Under the above conditions (118 °C, 30 min) when charging of all the reagents into the system at once, the PERO yield ranges from 55–57% (Table 2), while the epoxy group content changes in accordance with an excess of resorcinol, reaching a maximum value of 27% when the molar ratio of HCP:resorcinol is 1:24 (Table 2). A similar picture is observed with the gradual addition of alkali into the reaction mixture (Table 3), although with the HCP:resorcinol ratio 1:12, 1:16, 1:24, the overall yield of the oligomer and the epoxy group content increases significantly (by 10–20%). The latter indicates that with gradual loading of alkali, the rate of both direct interaction of epichlorohydrin with the –OH groups of resorcinol and the resulting hydroxyaryloxyphosphazenes, as well as the dehydrochlorination reaction of the resulting chlorohydrin groups, increases. Analysis of the ^1^H NMR spectra of the PERO (Figure 3) confirms the presence of chlorohydrin (CH) and small amounts of 2,3-dihydroxypropyl groups (DH) and 2-hydroxypropylene bridges in both fractions, in addition to the oxirane cycles. The presence of 2-hydroxypropylene bridges indicates the occurrence of interactions according to the scheme in Figure 4.

**Figure 5:**
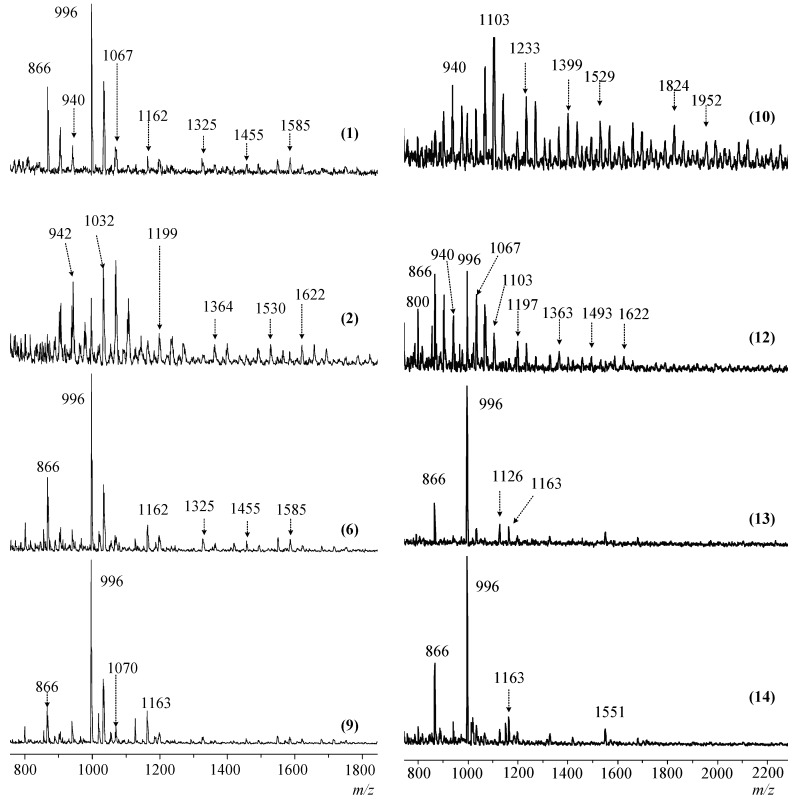
MALDI-TOF mass spectra of PERO synthesized at a molar ratio of HCP: resorcinol 1:16 (**1**, **6**, **13**), 1:12 (**2**, **12**) and 1:24 (**9**, **14**) at temperatures of 65 °C (**1**) and 116 °C (**2**–**14**), duration 90 min (**1**) and 30 min (**2**–**14**) and single-moment (**1**–**9**) and portioned (**10**–**14**) addition of NaOH. The numbers of the spectra correspond to the numbers of the samples according to the Table 2 and Table 3.

**Figure 6:**
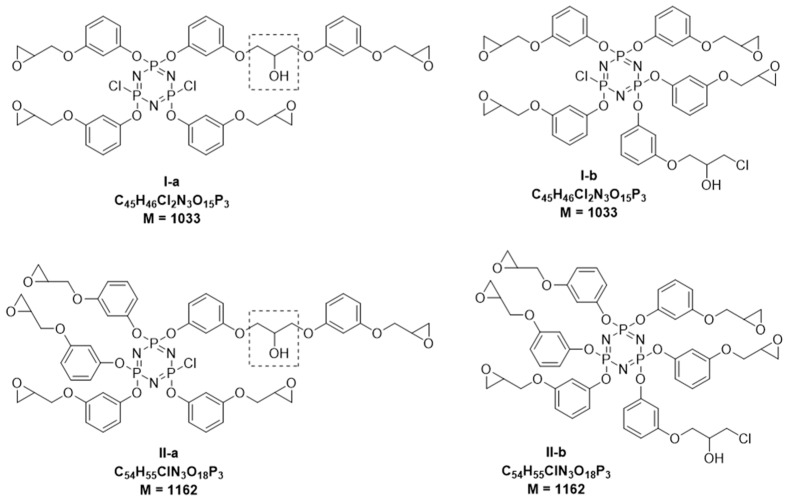
Structural isomers of epoxyphosphazenes with the same molecular weight, found on MALDI-TOF spectra.

The quantitative content of CG and DH groups in the PERO composition can be estimated from the MALDI-TOF mass spectra (Figure 5), the assignment of the main peaks to the assumed gross formulas of the compounds is given in Table 4 and Table 5. However, the incomplete dehydrochlorination and oligomerization of side chains lead to the formation of, among other compounds, a number of structural isomers having the same gross formulas, elemental composition, and molecular weight, but a different number of substituted chlorine atoms in the phosphazene cycle. The presence of these isomers in the PERO composition, for example, with a mass of 1033 in compounds I-a and I-b and 1162 in compounds II-a and II-b, as shown in Figure 6, complicates the interpretation of laser mass spectra.

According to ^1^H NMR spectra, the content of 2-hydroxypropylene bridges (circled in a rectangle in Figure 6) is small and depends little on the HCP:resorcinol ratio. The content of chlorohydrin groups is noticeably higher, which increases with a decrease in the amount of resorcinol by 1 mol of HCP. Taking into account the fact that the main components of the PERO are penta-substituted phosphazene cycles, it follows from the presented data that it is likely that compound I-b predominates among the isomers given.

The analysis of MALDI-TOF mass spectra also allows the establishment of the presence—in the composition of PERO—of an insignificant number of compounds whose molecules contain two partially substituted triphosphazene rings connected by a 1,3-dioxyphenylene bridge. The content of such compounds in most cases is 2–9% and reaches 40% only for products synthesized with the gradual loading of alkali and when the HCP:resorcinol molar ratio is 1:10. Table 5 shows the content of compounds with two phosphazene cycles calculated from MALDI-TOF-mass spectra.

With the seeming complexity of laser mass spectra and their apparent differences, the following points can be noted:

- more complex PERO compositions are formed at the molar ratios of HCP:resorcinol = 1:12, and there is greater presence of compounds whose molecules contain two triphosphazene cycles (*m*/*z* > 1200) in their composition;
- in the composition of the phosphazene fractions of oligomers synthesized at the HCP:resorcinol ratio > 1:12, there are mainly tetra- (*m*/*z* = 866) and penta-epoxy (*m*/*z* = 996) derivatives with content predominantly being of the latter;
- there is an insignificant amount or almost complete absence of hexa-(3-glicidyloxyphenoxy)-cyclotriphosphazene (*m*/*z* = 1126) in the composition of PERO;
- the most homogeneous composition of the phosphazene fraction is PERO synthesized at molar ratios of HCP:resorcinol = 1:16 and 1:24, especially in the case of gradual loading of solid alkali (Fig. 2, spectra 13 and 14). At a ratio of 1:24, predominantly tetra- (*m*/*z* = 866) and penta-substituted (*m*/*z* = 996) compounds are present in the phosphazene fraction.

Increasing the amount of diphenol above 24 moles per 1 mole of HCP does not lead to an increase in the degree of substitution of chlorine atoms in HCP. Even with the HCP:resorcinol ratio of 1:48, the main phosphazene-containing reaction product is still the penta-substituted phosphazene cycles with an insignificant content of tetra- and hexa-substituted cycles.

When the ratio of HCP:resorcinol is less than 1:12 (i.e., 1:8 or 1:10), the dehydrochlorination reaction is difficult, as evidenced by the increased content of chlorine and OH groups in the product. This fact is probably due to steric difficulties caused by the increase in the intensity of the gelation reaction. The latter is confirmed by the low yield of soluble oligomers and the high content of compounds with 2 and 3 phosphazene cycles in the soluble part of the product.

For the synthesis of PERO, it is preferable to use gradual (portioned) loading of alkali. The most optimal HCP:resorcinol ratio is in the range from 1:24 to 1:16, at which there is achieved: an acceptable phosphorus content of 3 to 5%, epoxy group content is 29-30%, and the minimum content of residual chlorine of 2.2–2.7%.

The values of viscosity PERO obtained at a ratio of HCP:resorcinol 1:16 and industrial bisphenol A based epoxy resin (such as diglycidyl ether of bisphenol A, DGEBA) is comparable (Table 6). At the same time, PERO viscosity is 10–20 times lower than that of phosphazene-containing epoxy resins based on bisphenol A with approximately the same content of the phosphazene component [76].

## 4. Conclusions

The synthesis of epoxyphosphazene-containing epoxy oligomers based on hexachlorocyclophosphazene and resorcinol in epichlorohydrin excess media, and with a portioned load of solid NaOH, allows the adjustment of the epoxy group content of the resulting oligomer to within 30% and the phosphorus content from 2 to 5%, by varying the ratio of HCP:diphenol. The viscosity of obtained resorcinol based epoxyphosphazene-containing resins is comparable to conventional bisphenol A based epoxies, and is much lower in comparison to similar epoxyphosphazene resins based on bisphenol A. Thus the obtained epoxyphosphazene resins may be used as a component of a binder for composite materials, adhesives, and paints.

## Figures and Tables

**Table 1:** The amounts of initial reagents for the synthesis of PERO.

The Molar Ratio of HCP:Resorcinol	Reagent Amount, g			Calculated Yield ^1^ (g)	Calculated Epoxy group Content ^1^ (%)
	HCP	Resorcinol	NaOH		
1:12	1.00	3.80	2.90	7.30	32.0
1:16	1.00	5.06	3.86	10.20	32.9
1:24	1.00	7.59	5.79	14.96	35.2

^1^ Here and in Table 2 and Table 3, the yield of the product, the content of epoxy groups, the phosphazene component, chlorine, and phosphorus were calculated assuming the formation of only the diglycidyl ether of resorcinol and penta-(3-glycidyloxyphenoxy)chlorocyclotriphosphazene.

**Table 2:** Reaction conditions of HCP, resorcinol, and epichlorohydrin, yield and epoxy group content of PERO with the charging of the entire amount of NaOH at the beginning of the reaction and molar ratio of HCP:resorcinol 1:16.

Experiment No.	Temperature (°C)	Reaction Time (min)	Product Yield (%)	Epoxy Group Content (wt.%)
1 ^1^	118	30	46	14.9
2	65 ^3^	90	58	19.9
3	90	30	71	15.8
4	100	30	55	20.2
5	118	15	75	21.6
6	118	30	69	22.2
7	118	45	75	21.4
8	118	60	68	21.9
9 ^2^	118	30	70	27.3

^1^ The molar ratio of HCP:resorcinol 1:12; ^2^ 1:24; ^3^ Synthesis conditions from patent [75].

**Table 3:** Reaction conditions of HCP, resorcinol and epichlorohydrin, the yield and composition of the products with portioned addition of NaOH. Reaction temperature 118 °C and duration 30 min.

Experiment No.	The Molar Ratio of HCP:Resorcinol	Product Yield (%)	Content (wt.%) ^1^				
			Epoxy Groups	–OH Groups	P	Cl	Phosphazene Component
10	1:8	61	5.5/28.5	5.2	6.8/5.6	10.8/2.6	73.1/60.0
11	1:10	71	14.5/30.6	4.1	4.8/4.4	8.3/2.3	51.3/47.3
12	1:12	77	21.0/32.0	2.2	4.0/3.7	4.4/2.1	42.5/39.8
13	1:16	89	28.6/32.9	2.0	3.0/2.7	2.4/1.9	31.6/29.0
14	1:24	90	29.6/35.2	0.6	2.0/1.8	1.9/1.7	21.2/19.1

^1^ found/calculated.

**Table 4:** Gross formulas of compounds with one triphosphazene cycle P_3_N_3_Cl_6-*n*_(OArOX)***_n_*** and their relative content in PERO according to MALDI-TOF mass spectrometry. Here and in Table 5, the designations of the radicals:

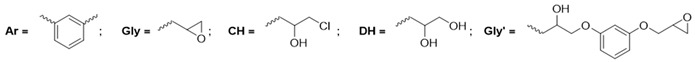											
The Number of Radicals X in the Gross Formula			Calculated Molecular Weight	The *m*/*z* Values of the Peaks on the MALDI-TOF Spectra of the PERO, in Parentheses—Peak Relative Intensity (%)							
				Portioned Addition of NaOH in Experiments No. from Table 3				Single-Moment Addition of All NaOH in Experiments No. from Table 2			
Gly	Gly’	CH		14	13	12	11	9	6	1	2
**Tetra-Substituted Cyclotriphosphazenes (*n* = 4)**											
4			867	867 (26.4)	866 (14.0)	866 (21.2)		866 (17.5)	866 (8.2)		866 (14.2)
3	1		1033		1033 (3.0)						
3		1	903			904 (9.5)	904 (3.7)			904 (5.1)	904 (8.5)
2		2	939			940 (7.2)	940 (5.5)		940 (3.5)	942 (6.0)	940 (5.0)
1	1	2	1106			1103 (4.9)	1103 (9.4)			1106 (6.1)	
1		3	976				976 (5.2)				
	1	3	1142				1141 (6.8)				
**Penta-Substituted Cyclotriphosphazenes (*n* = 5)**											
5			996	997 (65.0)	996 (75.1)	997 (12.5)		996 (40.9)	996 (45.1)	996 (10.0)	996 (29.4)
4	1		1162	1163 (3.9)	1163 (3.2)			1163 (6.7)	1163 (7.5)		1162 (3.5)
3	2		1329	1328 (0.6)		1325 (2.3)		1326 (1.5)	1326 (0.7)		1325 (2.5)
4		1	1033			1033 (10.6)	1032 (3.6)	1034 (21.5)	1034 (19.1)	1034 (27.3)	1032 (17.6)
3	1	1	1199					1197 (1.9)	1054 (2.9)	1199 (2.5)	1197 (3.7)
2	2	1	1365			1363 (3.2)	1363 (2.5)	1362 (0.9)		1364 (2.1)	1361 (1.0)
3		2	1069	1068 (0.9)		1067 (9.1)	1068 (7.5)		1070 (3.3)	1068 (28.5)	1067 (5.2)
2	1	2	1235			1233 (3.5)	1233 (6.3)			1233 (2.6)	
**Hexa-Substituted Cyclotriphosphazenes (*n* = 6)**											
6			1126	1127 (1.7)	1127 (2.8)				1127 (5.0)		
3	2	1	1495			1493 (0.8)	1491 (3.5)			1492 (2.2)	1491 (1.0)
4		2	1199			1197 (4.1)	1197 (3.4)				
Total relative content of compounds with one triphosphazene cycle ^1^ (%)				98.3	98.0	91.4	57.5	91.0	96.2	92.5	91.7

^1^ The rest are compounds containing two triphophazene rings connected with a 1,3-dioxyphenylene bridge.

**Table 5:** Compounds with two triphosphazene cycles contained in the reaction product of HCP, resorcinol and epichlorohydrin (experiment No. 11, Table 3) with gross formula P_3_N_3_Cl_5−*n*_(OArOX)*_n_*-OArO-P_3_N_3_Cl_5−*m*_ (OArOX)*_m_*.

*n*	*m*	The Value and the Number of X Radicals in the Gross Formula ^1^				Calculated Molecular Weight	Peaks on MALDI-TOF Spectra	
		Gly	Gly’	CH	DH		*m*/*z*	Relative Intensity (%)
2	2	3			1	1269	1269	6.0
3	2	4			1	1399	1399	4.9
3	2	3		1	1	1436	1435	3.8
3	3	5			1	1529	1529	3.8
3	3	4		1	1	1565	1566	1.7
3	3	3		3		1620	1621	1.5
4	3	6			1	1659	1658	3.3
4	3	4	2			1991	1988	2.4
4	3	5		1	1	1695	1693	2.8
4	3	4	1	1	1	1861	1860	1.6
4	3	2	2	3		2082	2081	1.6
4	3	4		2	1	1731	1731	1.5
4	4	7			1	1788	1788	0.9
4	4	6	1		1	1954	1952	2.5
4	4	6		1	1	1825	1824	1.7
-	-	4	1		1	2116 ^2^	2117	1.3
-	-	5	1		1	2246 ^2^	2246	1.2

^1^ The distribution of residual chlorine in cycles is conditional ^2^ Compounds with three phosphazene cycles.

**Table 6:** The values of viscosity PERO obtained at a ratio of HCP:resorcinol 1:16.

Resin Type	The Values of Viscosity (Pa∙s) at the Temperature of (°C)				
	20	40	50	60	70
DGEBA	5.83	0.86	0.32	0.13	0.06
PEO based on bishenol A [76]	-	130	25	6	2
PERO (experiment No. 13, Table 3)	2.43	1.94	0.66	0.29	0.15
